# PAIVS: prediction of avian influenza virus subtype

**DOI:** 10.5808/GI.2020.18.1.e5

**Published:** 2020-03-31

**Authors:** Hyeon-Chun Park, Juyoun Shin, Sung-Min Cho, Shinseok Kang, Yeun-Jun Chung, Seung-Hyun Jung

**Affiliations:** 1 Department of Biomedicine & Health Sciences, College of Medicine, The Catholic University of Korea, Seoul 06591, Korea; 2 Department of Microbiology, College of Medicine, The Catholic University of Korea, Seoul 06591, Korea; 3 Integrated Research Center for Genome Polymorphism, College of Medicine, The Catholic University of Korea, Seoul 06591, Korea; 4 Chungbuk Veterinary Service Laboratory, Chungju 27336, Korea; 5 Cancer Evolution Research Center, College of Medicine, The Catholic University of Korea, Seoul 06591, Korea; 6 Department of Biochemistry, College of Medicine, The Catholic University of Korea, Seoul 06591, Korea

**Keywords:** AIV subtypes, avian influenza virus, next-generation sequencing, viral genome

## Abstract

Highly pathogenic avian influenza (HPAI) viruses have caused severe respiratory disease and death in poultry and human beings. Although most of the avian influenza viruses (AIVs) are of low pathogenicity and cause mild infections in birds, some subtypes including hemagglutinin H5 and H7 subtype cause HPAI. Therefore, sensitive and accurate subtyping of AIV is important to prepare and prevent for the spread of HPAI. Next-generation sequencing (NGS) can analyze the full-length sequence information of entire AIV genome at once, so this technology is becoming a more common in detecting AIVs and predicting subtypes. However, an analysis pipeline of NGS-based AIV sequencing data, including AIV subtyping, has not yet been established. Here, in order to support the pre-processing of NGS data and its interpretation, we developed a user-friendly tool, named prediction of avian influenza virus subtype (PAIVS). PAIVS has multiple functions that support the pre-processing of NGS data, reference-guided AIV subtyping, de novo assembly, variant calling and identifying the closest full-length sequences by BLAST, and provide the graphical summary to the end users.

**Availability:** PAIVS is available at http://ircgp.com/paivs.

## Introduction

The avian influenza virus (AIV) genome comprises eight segments (polymerase basic 2, polymerase basic 1, polymerase acidic, hemagglutinin [HA], nucleoprotein, neuraminidase [NA], matrix, nonstructural) that encoded up to 11 proteins [1]. Based on the viral surface glycoproteins HA and NA, AIVs are divided into various subtypes. To date, there are 18 different HA subtypes and 11 different NA subtypes (H1 through H18 and N1 through N11, respectively) [2], which potentially form 144 HA and NA combinations by genetic reassortment. Although most of them are of low pathogenicity and cause mild infections in birds, some AIV combinations including HA H5 and H7 cause highly pathogenic avian influenza (HPAI), which is characterized by high morbidity and mortality [3,4]. Indeed, large HPAI outbreaks in domestic poultry occurred during 2014–2018 in South Korea. The outbreaks of the 2016–2017 and 2017–2018 winter seasons, caused by novel reassortant clade 2.3.4.4 H5N6 viruses, resulted in loss of one billion birds in 440 farms in South Korea (https://www.kahis.go.kr/). Therefore, accurate and rapid subtyping of AIV is important to prepare and prevent for the spread of HPAI.

There are several methods to detect the AIVs such as rapid influenza diagnostic test (RIDT), nucleic acid-based tests (NATs), and next-generation sequencing (NGS) [5]. RIDTs, the most widely used method, can detect AIVs rapidly but do not provide information on AIV subtype or specific virus strain information. NATs, which include reverse transcriptase-PCR and loop-mediated isothermal amplification-based assay, detect virus-specific DNA or RNA sequences. Although most NATs are more sensitive and specific than RIDTs, they are laborious and time-consuming tests to get subtype information. In contrast to these methods, NGS can analyze the full-length sequence information of all eight AIV segments at once, so it can identify subtypes sensitively and accurately. Furthermore, since single nucleotide variants and evolutionary analyses are possible with the complete genome sequencing data, there are many advantages to identify the origin and pathogenicity of AIVs. However, an analysis pipeline of NGS-based AIV sequencing data, including AIV subtyping, has not yet been established.

In this study, we developed a user-friendly tool, named prediction of avian influenza virus subtype (PAIVS), to support the pre-processing of NGS data and its interpretation. PAIVS analyzes the NGS data for pre-processing, reference-guided AIV subtyping, de novo assembly and variant calling and provides graphical summary for subtype identification. In addition, PAIVS supports the BLAST (Basic Local Alignment Search Tool) function to identify the closest full-length sequences that can be used as genetic resources for downstream analysis, such as phylogenetic analysis.

## Overview Pipeline

**Fig. 1.**
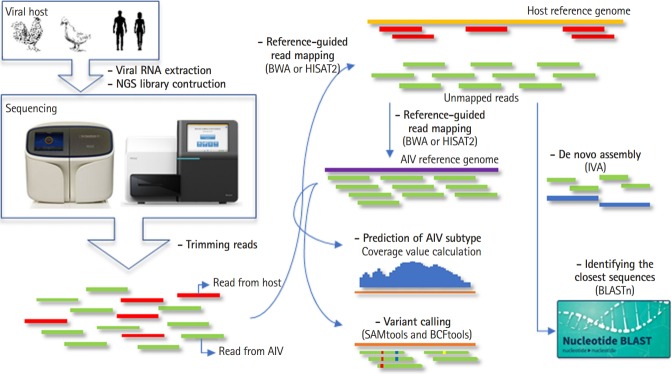
Workflow of prediction of avian influenza virus subtype (PAIVS). PAIVS is an automated pipeline that analyzes avian influenza virus (AIV) next-generation sequencing (NGS) data and consists of five steps; pre-processing, reference-guided alignment or de novo assembly, subtyping, variant calling, and identifying the closest sequences. First, PAIVS takes FASTQ files from paired-end viral genome sequencing data as input. In the pre-processing step, PAIVS trims the sequence reads to remove the sequences with adaptor or low base quality. Trimed reads are then aligned to the host reference genome, such as avian or human, to remove the sequences from the host. In the next step, unmapped reads are aligned to the AIV reference genome and subsequently identify subtypes and variants based on the sequence composition and coverage. Users can also select de novo assembly option to get the closest full-length sequences and use them for downstream analysis. IVA, Iterative Virus Assembler.

PAIVS is an automated pipeline that analyzes AIV NGS data and consists of five steps; pre-processing, reference-guided alignment or de novo assembly, subtyping, variant calling, and identifying the closest sequences. We implemented PAIVS using python and its workflow is illustrated in Fig. 1. First, PAIVS takes FASTQ files from paired-end viral genome sequencing data as input. In the pre-processing step, PAIVS trims the sequence reads to remove the sequences with adaptor or low base quality. Trimed reads are then aligned to the host reference genome, such as avian or human, to remove the sequences from the host. In the next step, unmapped reads are aligned to the AIV reference genome and subsequently identify subtypes and variants based on the sequence composition and coverage. Users can also select de novo assembly option to get the closest full-length sequences and use them for downstream analysis. All results for each step is stored in its own directiry.

## Data Pre-processing

In the pre-processing step, TRIMMOMATIC [6] tool trims the sequence reads to remove the sequences with adaptor or low base quality. Host reads subtraction by read mapping is performed by using the HISAT2 [7] or BWA [8] aligner against host organism genome. SAMtools software package [9] ‘view’ option is used for extracting unmapped read. Unmapped viral reads are then mapped to the AIV reference genome using the HISAT2 or BWA aligner. When we compared the mapping rate, memory usage, and run time between two aligners, BWA mem algorithm showed resonable run time and mapping rate to the reference AIV genome (Supplementary Table 1). HISAT2 was faster and used fewer resources than BWA, but more than 90% of sequencing reads were not mapped to the AIV genome. Therefore, we recommend to use BWA aligner unless user has ultra high depth sequencing data (>10,000×).

## Prediction of AIV Subtype

In order to predict the AIV subtypes, PAIVS calculates the segment mean coverages as a coverage value for each 18 different HA and 11 different NA segments by using SAMtools. Coverage value means the sum of the coverages is then normalized by dividing by the total length of segment.

Segment Mean Coverages=∑k=1pDpp

where *D_p_* is depth of each genomic position, *p* is segment length, *k*=1,…, *p* is each genomic position.

**Fig. 2.**
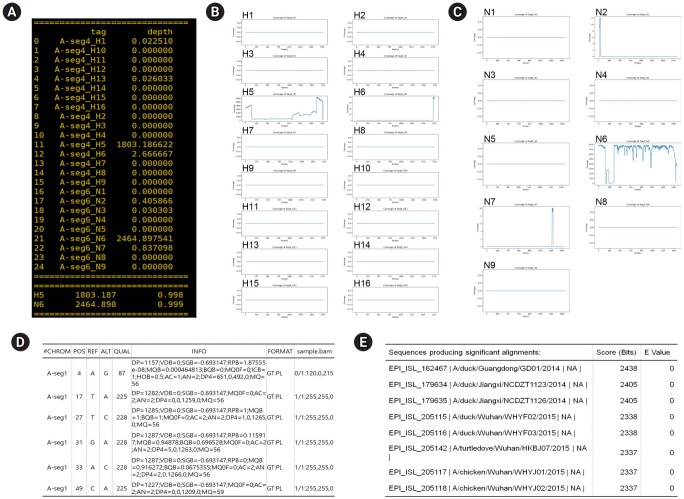
Examples of prediction of avian influenza virus subtype (PAIVS) output. To evaluate the PAIVS, we analyzed a H5N6 avian influenza virus (AIV) confirmed by PCR and Sanger sequencing test. For the reference-guided read mapping, AIV genome sequences were obtained from Influenza Virus Resource Database (https://www.ncbi.nlm.nih.gov/genomes/FLU) and customized to analyze 16 different hemagglutinin (HA) and 9 different neuraminidase (NA) segments (H1 through H16 and N1 through N9). (A) PAIVS generates the coverage value for each HA and NA segments and presents the predicted subtype. As expected, analyzed sample was predicted as H5N6 subtype. (B, C) PAIVS also generates image files for coverage depth of HA (B) and NA (C) segments. Coverage depth of H5 and N6 segments was much higher than other segments. (D) Detected single nucleotide variants are reported as a variant call format. (E) The sequences closest to the analyzed segment are reported with BLAST score and E-value.

The coverage values are displayd on the linux terminal console and saved a comma separate text file (Fig. 2A). In addition, coverage depth for each HA and NA segments is saved as a image file in png format (Fig. 2B and 2C).

## Variants Calling and De Novo Assembly

SAMtools and BCFtools [10] are implemented in PAIVS to detect the single nucleotide variant, which are known to be faster and use less memory than other variant calling methods such as Varscan2 [11] and HaplotypeCaller [12]. The result is saved as a text file in variant call format, an example is shown in Fig. 2. Regarding de novo assembly, Iterative Virus Assembler (IVA) designed to assemble virus genomes [13] is implemented in PAIVS. When the viral FASTA file is generated as an output of the IVA, the closest full-length sequences are then identified by BLAST [14] (Fig. 2E). Both variants and BLAST results can be used as genetic resources for further downstream analysis, such as phylogenetic analysis and clade classification.

## Conclusion

In this paper, we introduce the PAIVS which is a user-friendly tool with multiple functions that support the pre-processing of NGS data, reference-guided AIV subtyping, de novo assembly, variant calling, and identifying the closest full-length sequences by BLAST and provide the graphical summary to the end users. In addition, PAIVS can be applied to other vial genomes for viral genome detection. By replacing the reference genome in the configuration file, users can easily perform read mapping, variant calling, de novo assembly, and identifying the closest full-length sequences with BLAST. However, the viral subtyping and graphical summary functions of PAIVS were specifically designed for AIVs, so there is a limit to applying these functions to other viral genome. Considering that NGS technology is becoming a more common for detecting AIVs and predicting subtypes, PAIVS can be helpful for beginners who are not familiar with NGS-based AIV data processing. PAVIS is freely available at http://ircgp.com/paivs.

## Supplementary Materials

Supplementary Table 1. Comparison of the mapping rate, memory usage, and run time between HISAT2 and BWA aligner
