# Supplementary material for: PAIVS: prediction of avian influenza virus subtype
Source: Genomics Inform. 2020 Mar 31;18(1):e5. doi: 10.5808/GI.2020.18.1.e5 (PMC7120348; doi:10.5808/GI.2020.18.1.e5)
Supplement: Supplementary Table 1. — Comparison of the mapping rate, memory usage, and run time between HISAT2 and BWA aligner [file gi-2020-18-1-e5-supple1.docx]

**Supplementary Table 1.** Comparison of the mapping rate, memory usage, and run time between HISAT2 and BWA aligner

|  | Run time | | | | | | Memory usage (KB) | | Mapping rates (%) | |
| --- | --- | --- | --- | --- | --- | --- | --- | --- | --- | --- |
|  | HISAT2 | | | BWA (MEM) | | | HIAST2 | BWA (MEM) | HIAST2 | BWA (MEM) |
|  | Thread 2 | Thread 4 | Thread 8 | Thread 2 | Thread 4 | Thread 8 |  |  |  |  |
| S1 | 0m34.720s | 0m16.868s | 0m17.834s | 1m20.332s | 0m42.727s | 0m31.437s | 884 | 418,008 | 0.19 | 88.87 |
| S2 | 0m55.342s | 0m13.532s | 0m13.394s | 1m35.105s | 0m53.374s | 0m36.142s | 888 | 411,920 | 0.26 | 93.23 |
| S3 | 0m48.453s | 0m12.527s | 0m13.232s | 2m21.670s | 1m17.922s | 0m42.026s | 856 | 388,024 | 0.23 | 94.62 |
| S4 | 0m42.657s | 0m13.654s | 0m14.310s | 1m26.063s | 0m47.207s | 0m28.993s | 856 | 266,668 | 0.16 | 89.66 |
| S5 | 0m40.107s | 0m17.305s | 0m14.020s | 0m38.776s | 0m23.869s | 0m15.906s | 888 | 421,844 | 0.15 | 90.34 |
